# Ovarian adult stem cells: hope or pitfall?

**DOI:** 10.1186/1757-2215-7-71

**Published:** 2014-07-04

**Authors:** Ancuta Augustina Gheorghisan-Galateanu, Mihail Eugen Hinescu, Ana Maria Enciu

**Affiliations:** 1 Department of Cellular and Molecular Medicine, Carol Davila University of Medicine and Pharmacy, 8 Eroii Sanitari Blvd., 050474 Bucharest, Romania; 2 C.I.Parhon National Institute of Endocrinology, 8 Eroii Sanitari Blvd., 050474 Bucharest, Romania; 3 V.Babes National Institute of Pathology, 8 Eroii Sanitari Blvd., 050474 Bucharest, Romania

**Keywords:** Ovarian stem cells, Adult oogenesis, Human ovary, Adult stem cells, Oocytes, Fertility

## Abstract

For many years, ovarian biology has been based on the dogma that oocytes reserve in female mammals included a finite number, established before or at birth and it is determined by the number and quality of primordial follicles developed during the neonatal period. The restricted supply of oocytes in adult female mammals has been disputed in recent years by supporters of postnatal neo-oogenesis. Recent experimental data showed that ovarian surface epithelium and cortical tissue from both mouse and human were proved to contain very low proportion of cells able to propagate themselves, but also to generate immature oocytes in vitro or in vivo, when transplanted into immunodeficient mice ovaries. By mentioning several landmarks of ovarian stem cell reserve and addressing the exciting perspective of translation into clinical practice as treatment for infertility pathologies, the purpose of this article is to review the knowledge about adult mammalian ovarian stem cells, a topic that, since the first approach quickly attracted the attention of both the scientific media and patients.

## Introduction

The human ovary is responsible for providing mature and competent oocytes for reproduction. In addition, it is responsible for the secretion of various hormones and growth factors and cytokines that are involved in signaling pathways of folliculogenesis and oogenesis.

Many years, ovarian biology has been based on the principle (dogma) that oocytes reserve in female mammals included a finite number established before or at birth and it is determined by the number and quality of primordial follicles developed during the neonatal period. The restricted supply of oocytes in adult female mammals has been disputed in recent years by supporters of neo-oogenesis. This new threatening-dogma perspective states that renewable germline stem cells (GSCs) are present in the postnatal mammalian ovary.

The supporters of neo-oogenesis claim the existence of GSCs in the ovarian surface epithelium (OSE) or the bone marrow (BM) and peripheral blood, which can differentiate in the ovary into oocyte, granulosa phenotype, fibroblast-like cells and in vitro, under appropriate stimulation, in neural and on mesenchymal type cells. Endpoints ranging from oocyte counts to genetic lineage tracing and transplantation experiments support a paradigm shift in reproductive biology involving active renewal of oocyte-containing follicles during postnatal life [1]. Although such ovarian GSCs are well characterized in non-mammalian model organisms, the findings that support the existence of adult ovarian GSCs in mammals have been met with considerable evidence that disputes their existence [2]. Adult ovary contains cellular subpopulations displaying stem cell markers such as c-kit [3] or Oct4 [4]. The best characterized stem cell population and stem cell niche is the germline stem cell in the Drosophila ovary (reviewed in [5]), but recently other types of stem cells have been described in the adult ovary, such as very small embryonic-like stem cells [6] or a subpopulation of granulosa cells [7].

The reports about existence and potency of adult mammalian ovarian stem cells yielded controversies in the scientific media, like most new and groundbreaking reports. The purpose of this article is to review the knowledge about adult mammalian ovarian stem cells, both pro and con opinions on a topic that, since the first approach, quickly attracted the attention of both the media and patients.

## Review

### 1. Mammalian ovarian germline stem cells

#### 1.1. Histological structure of the ovary

When assessing a cell population, one should first refer to the histological organization of the studied structure, in an attempt to identify the different pools of putative stem cells.

**Figure 1:**
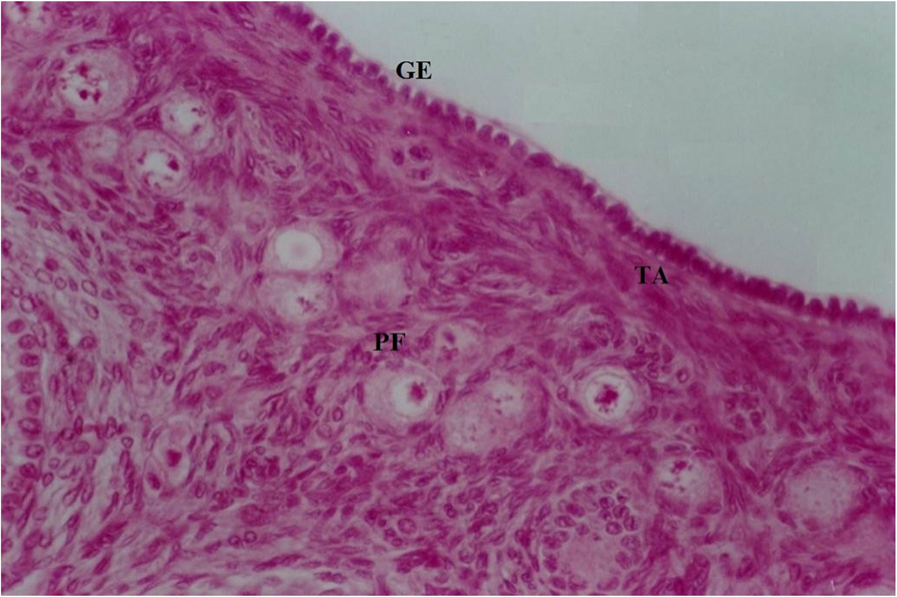
**Structure of the ovary.** The cortical region is surrounded by a simple epithelium classically named germinative epithelium (GE). Underlying the ovarian surface epithelium is a connective tissue layer, the tunica albuginea (TA). Groups of primordial follicles (PF) are present in the ovarian stroma. Col HE stain x40.

The female gonad is organized as a parenchymatous organ, with an outer, cortical region and an inner medullar one (Figure 1). It is covered by a simple epithelium, classically named germinative epithelium, which, occasionally, may invaginate into the ovarian cortex, forming epithelial cords, or if the link with the surface is severed, cysts. Interestingly, the ovarian surface epithelium (OSE) cells were proven to undergo an epithelial-mesenchymal transition in vitro, that can be reversed under proper stimuli [8]. Under OSE is albuginea, a connective tissue rich in collagen and reticular fibers, along with fibroblasts and fibrocytes. Underneath lies the cortical zone, a dense connective tissue stroma that harbors ovarian follicles in various stages of development, each containing an oocyte. Ovarian tissue is rich in primordial follicles, which make up the majority of the total follicular population in the human ovary. Besides oocytes, at least another two types of cells are present in the cortical zone: i) epithelial cells of granular layer of ovarian follicles and of endothelium of blood vessels; ii) fixed or migrated connective tissue cells (e.g. fibroblasts, fibrocytes, inner and outer theca cells, and macrophages, respectively [9]. The origin of these types of cells resides in correspondent precursor cells, which, interestingly, are different for ovarian follicular cells, otherwise functionally interdependent: granulosa cells and theca cells. While the latter are recruited and further differentiated from the ovarian stroma under granulosa-c-kit ligand signaling [10], for granulosa cells there are evidence suggesting common origin with surface epithelial cells, starting with commune embryonic origin in coelomic epithelium [11]. Today, there is a putative developmental relationship between them, as discussed further. Over the lifespan of the organism, ovarian aging occurs inevitably and it is characterized by both a reduction in eggs quality and a drastic reduction in the total number of ovarian follicles [12]. In mammals, primordial follicles serve as the source of developing follicles and oocytes during the entire reproductive life of the organism. As a woman ages, her pool of primordial follicles gradually shrinks and menopause occurs when the number of primordial follicles falls to below about 1000 [13]. The reduction of oocyte quality with aging is believed to be mainly due to an increase in meiotic nondisjunction that leads to an increased rate of aneuploidy in the early embryos [14].

#### 1.2. Origin of mammalian germline stem cells

Primordial germline stem cells (PGSs) are of extraovarian origin, migrated in the ovaries during embryonic development. In humans, PGSs originate in the posterior epiblast cells, under the signaling influences of BMP-family morphogens [15]. By weeks 4-5, PGSs are traceable in the extraembryonic mesoderm of the wall of the yolk sac from where they will migrate and populate the urogenital ridge and further differentiate into oogonia. Through mitotic divisions, the number of oogonia increases until the fifth month of intrauterine life, when the first meiotic division is triggered and the oogonia become oocytes.

The differentiating potential of PGS in vitro is well characterized so far and involves several directions: i) differentiation in mature gametocytes; ii) undifferentiation into embryonic stem cells [16]; iii) transdifferentiation into hematopoietic progenitor cells [17].

In the last few years, some authors reported PGSs originating in the OSE of fetal gonads and give rise to secondary germ cells and primitive granulosa cells [18]. The adult ovary also apparently retains the ability to generate germ cells from ovarian precursors. Putative germline stem cells (GSC) should share several features with other adult stem cells: i) stemness markers such as Oct4, Nanog, c-kit ii) slow-dividing and hence, long-retaining synthetic nucleosides such as BrdU; iii) asymmetric division capacity and renewal of its own pool; iv) multipotency and the capability to form in vitro, or upon transplantation, all the related cell populations. The results reported so far, used several markers to identify ovarian germline stem cells, such as pluripotency markers as Oct4, stage-specific embryonic antigen-4 (SSEA-4) [6], telomerase activity and germline markers, markers whose expression changes as differentiate towards a mature phenotype. A hallmark of the germline that has been overlooked so far is suppression of the somatic program of gene expression and in seeking evidence of imprinting status a larger number of genes should be examined to verify that epigenetic reprogramming has been executed appropriately [19]. Some groups advanced the idea of an extraovarian origin [20], which others endorsed only under pathological circumstances [21]. Using as a starting point the common origin for both GSC and hematopoietic stem cells (HSC) – the proximal epiblast – Johnson et al. searched for germline precursors in the bone marrow, using as identification tag several germline markers such as Mvh, Dazl, Stella Fragilis or Nobox. Their selection finally generated a bone marrow-derived GSC phenotype containing both line-specific and stem-specific markers: Mvh+/lin-/Sca 1-/c-kit + [20]. They further attempt to restore follicular population of chemotherapy- depleted ovaries by bone marrow transplant or peripheral blood cell transplantation. However, if the presence of Mvh in the bone marrow is a strong witness for their plea, there are reports of transdifferentiated HSC, either from bone marrow donors or own circulating HSC, into adult stem cells of other injured organs (e.g. lung, [22] liver [23], heart [24]). Without previous aggression (chemotherapy, irradiation) however, BM transplant does not seems to contribute to GSC ovarian pool [25].

During the last few years, several groups reported the presence of very small, embryonic like pluripotent stem cells in various tissues [26,27], including adult ovary [28-30]. Very small embryonic-like cells (VSELs) are 3.5 μm in diameter, diploid cells that express markers of pluripotency, such as Nanog, Klf-4, SSEA-4 [31]. VSELs were isolated from ovarian surface epithelium and were shown to expresse early embryonic developmental markers such as stage-specific embryonic antigen-4 and Oct-4, Nanog, Sox-2, and c-kit [32]. In vitro they formed all three embryonic layers, but upon transplantation in immunodeficient mice, they failed to generate teratomas [29]. In ovaries, they have been identified between the OSE cells and similar to OGS, they seem able to generate in vitro oocyte-like cells [30]. To better gain a comprehensible understanding on the origin of both VSELs and OGS and possibly on the relationship between them, a gene expression profiling would provide more insight. However, since both cell types have just been identified and described, several profiling experiments use no precursor cells, but oocyte-like derived cells, obtained from the putative precursors in cell culture [33].

#### 1.3. Germline stem cell niche

Accepting the idea of an adult germline stem cell, one must also try to define the stem cell niche, which, for other defined adult stem cells comprises several compartments: i) a slow-dividing adult stem cell population; ii) a rapid proliferating compartment of precursors cells – transit amplifying cells (TA cells); iii) supportive cells, usually of mesenchimal type. A part from the cellular partners, extracellular matrix [34] and gradients of signaling pathway ligands [35] also play important roles in niche maintenance. The best described ovarian niche is undoubtedly in Drosophila ovary, where the presence of GSCs is well established [36].

There are several arguments that both fetal ovary and postnatal ovary are functionally compartmentalized to ensure proper oocyte development. The correct spatial organization and proper neighboring relations between ovarian fetal cells are required during in utero gonad development to generate follicles, as proven by Nicholas et al. In their experiment, they attempt to “reassemble” mouse ovarian tissue from single cell suspensions, in order to further obtain folliculogenesis upon transplantation into depleted ovaries. Until mid-gestation (d13.5), their attempts were unsuccessful. However, once a certain developmental stage has been reached, isolation of cells and in vitro re-arrangement was sufficient to further generate ovarian follicles [37]. Bukovsky argues that embryonic ovarian surface epithelium is established early during development and involves vascular pericytes and immune cells [38].

Furthermore, the adult ovarian cortex provides the inhibitory stimuli needed to maintain the oocyte from completing its cell cycle and there are several maturation triggers known so far to induce the completion of meiosis I: i) low cyclic adenosine monophosphate (cAMP); ii) members of Epidermal Growth Factor (EGF) family, such as amphiregulin and ephiregulin [39]; iii) possibly, the follicular fluid-derived meiosis-activating sterol (FF-MAS) – an intermediate along the biosynthetic pathway from lanosterol to cholesterol; iv) steroids such as testosterone or estradiol [40].

There are reports, however, of in vitro derived GSC from embryonic stem cells under appropriate stimuli by enriched growth media and cultivation on fibroblast feeder layer [41]. Such a manufactured growth environment is surely not similar to an in vivo model of stem cell niche, but it appears to provide all the necessary clues.

Taking into consideration the hypothesis of GCS being harbored in the OSE, implicitly a niche must exist, but it has not yet been described. Very recently, Flesken-Nikitin et al. advanced the idea that hilum region of the mouse ovary harbors a putative niche for the OSE [42].

Along with the postnatal oogenesis hypothesis, also arises the idea that menopause may actually be the result of a compromised somatic niche [43,44]. Experiments demonstrating the presence of stem cells in peri-menopausal ovary and their increase after FSH treatment suggests that stem cells retain the ability to proliferate, but menopause sets in due to a compromised microenvironment which does not allow the stem cells to differentiate and assemble into primordial follicles [45].

### 2. Ovarian germline stem cells (Ogs) experimental data

The multipotency of a stem cell may be demonstrated through several types of experiments: i) specific surface receptors expression analysis that changes along differentiation pathway; ii) generation of lineage-specific cells in vitro; iii) repopulation of a depleted organ in vivo (e.g. repopulation of bone marrow after prelethal irradiation). Such experimental approaches were used for putative adult ovarian germline stem cells, in order to prove their presence and their regenerative abilities. To identify and further characterize OGS, OSE or cortical tissue from mouse and human were used as putative pools. Indeed, both sources were proved to contain in very low proportion cells able to propagate themselves but also to generate immature oocytes in vitro or in vivo- when transplanted into mouse ovaries [46].

Furthermore, recent data argued for their multipotency, when putative ovarian stem cells were successfully differentiated into cells of the three primary germ layers [47].

If the faith of these cells is, by now, proved and accepted to be towards differentiation downstream female germline, their origin is still a controversy. Among the first groups to promote postnatal folliculogenesis, Tilly’s group also proposed at first a bone marrow origin for the putative stem cells [20,21]. Bukovsky et. al endorsed the idea of OSE harboring bipotent cells to give rise to secondary germ stem cells and primitive granulosa cells [48]. Recent data advanced the idea that OGS could be very small embryonic-like stem cells - descendants of epiblast stage pluripotent stem cells, deposited in various body organs including the gonads in early stages of development, as a quiescent stem cell population [29]. However, transplantation of cultivated OGS into immunodefficient mice did not result in teratoma formation (assay used to assess pluripotency for stem cells) [49], nor did transplantation of putative human ovarian stem cells, which are probably not of a germline origin [47]. On a contrary, Gong et al. reported that their ESC-like cells isolated from mouse ovaries formed terotoma when transplanted in to SCID mice [50].

The last four years were very fruitful in terms of establishing cell culturing conditions for germline precursor cells. Several independent laboratories have reported successful protocols of female germline stem cells culture initiation and subsequent yielding of immature oocytes [1], as discussed in the following section.

#### 2.1 Timeline of in vitro OGS experiments

##### 2. 1.1. Laboratory animal models

Maybe not first, but perhaps most acknowledged landmark in OGS is 2004 Tilly’s research group report on postnatal follicular renewal in mouse ovary, from a population of BrdU/mouse VASA homologue (MVH) positive cells, located in the OSE [51]. They also demonstrated the existence of adult folliculogenesis by implanting GFP-positive ovarian grafts in wild type adult, fertile mouse ovaries. After 3-4 weeks, recipient ovaries exhibited new follicles, with GFP-positive oocytes and wild-type granulosa cells.

In 2006, Kerr et al. demonstrated by unbiased stereological methods quantifying both healthy and atretic follicles, that mean primordial follicle numbers per ovary did not decline significantly in postnatal mouse ovaries [52].

During the next few years, “ovarian stem cells” were mostly in a gray zone, the literature providing mostly interesting debates on their existence [53-55].

In 2009, Zou et al. isolated double positive BrdU/MVH cells from the ovarian surface epithelium of new born and adult mice that were further maintained in culture for six months of more. The cells expressed a protein phenotype of stemness (Oct 4, Nanog and high telomerase activity) and no markers of meiotic activity or further differentiation down germline lineage [56]. One year later, Pacchiarotti et al. also reported maintenance in cell culture of ovarian Oct4 + cells for more than a year and added to previous knowledge, data about localization outside follicular environment, characterization and multipotency [49].

##### 2.1.2 Human ovarian tissue

In 2004 Bukovsky et al. proposed that adult human ovaries contain germline putative stem cells in the OSE and ovarian cortex, as proven by imagistic studies and immunohistochemistry for zona pellucida proteins (ZPPs) such as PS1 [8]. Cytokeratins (CK) expression was also used to differentiate between epithelial, CK + cells (surface cuboidal cells, granulosa cells) and non-epithelial, CK- cells (mesenchymal, oocytes) and to advance the idea of bipotential stem cells that may generate primitive granulosa cells and primitive germ cells. Bukovsky et al. continued their line of investigation and reported in vitro identification of granulosa cells and oocytes-like cells in human OSE-derived cell cultures. Furhermore, it is worth noting that they used different culturing conditions for each of their experiments, hence the variety of reported results [57]. Further inquiry of OSE through immunohistochemistry of nuclear division (Ki - 67) or meiotic markers by Liu et al. returned negative, as did the c-kit staining. c-kit was, however, present in the granular cells of primordial and primary follicles, along with Oct4. The transcription factor, known as a pluripotency marker, was not present in surface epithelium or tunica albuginea [58]. Virant Klun’s group continued Bukovsky line of investigation on postmenopausal women and an OSE cell culture was successfully set-up in all cases. During the first 3 weeks of culture, oocyte-like cells grew up to 95 μm, expressing Oct-4A and Oct-4B, c-kit, VASA and ZP2, but not SCP3 [32].

The same group continued their work in 2009, isolated putative stem cells from OSE scrapings in 21 postmenopausal women with no naturally present follicles and oocytes, that further maintained in cell culture. OSE cell culture spontaneously developed oocyte-like cells [59]. Virant-Klun et al. furhter isolated 2-4 μm cells from OSE that expressed early embryonic developmental markers and some developed into oocyte-like cells under appropriate stimulation [59]. Molecular analysis of these small cells revealed epigenetic characteristics similar to epiblast/migrating PGC-like (epigenetic reprogramming profiles of Oct4, Nanog and Stella loci and unique patterns of genomic imprinting) [60]. Cell cultures of enzymatically digested human ovarian cortex also contain small embryonic like cells, SSEA-4+/Oct4+, out of which a small subpopulation was VASA + and based on microarray data on gene expression profiling, Virant-Klun et al. proposed them as putative stem cells [30].

In 2012, based on accumulated knowledge, White et al. optimized the protocol of OSE isolation from patients and established a cell culture that spontaneously generated oocytes. When injected in NOD-SCID mice ovaries, they generated primordial and primary follicles [46].

At the same time, using a lineage-tracing experiment based on DDX4 (DEAD box polypeptide 4), also known as Ddx4 or Mvh (mouse VASA homolog) in mice, Zhang et al. showed that the Ddx4-expressing cells from postnatal mouse ovaries did not enter mitosis, nor did they contribute to oocytes during de novo folliculogenesis [61], arguing against experimental results proposed by Zou [62] and White [46].

#### 2.2 In vivo mammalian models

From the beginning it should be mentioned that, as there is a lack of ovarian tissue available for research, human reports are scarce.

In vivo results are intricately related to in vitro research, especially when transplantation studies involve cell suspensions. In order to correctly interpret the results, one must properly isolate the cell population to be transplanted. More accurate, perhaps, are transplantation models involving the entire postnatal ovary, that would allow tracing of newly generated germ cells. Some experimental models followed the lessons from hematopoietic stem cells research, attempting repopulation of a depleted organ by putative multipotent cells. For gonads, experimental depletion was achieved chemically, through busulphan treatment. Such an approach was used by Johnson et al., who reported postnatal folliculogenesis after elimination of primordial follicles with busulphan treatment. They transplanted wild-type ovaries into transgenic mice expressing green fluorescent protein and reported preantral follicles with GFP negative granulosa cells and GFP positive oocytes [51]. Also following busulphan treatment, Zou et al. repopulated depleted mice ovaries with GFP positive, cell culture-selected Oct4 positive cells. Beside the repopulation of ovaries with follicles containing GFP-positive oocytes, they also reported successful mating and GFP- bearing offsprings [56].

Nicholas et al. transplanted mouse fetal ovarian tissue (dissociated to single cell suspension and re-aggregated) into ovarectomized, immunodeficient mice and reported posttransplant folliculogenesis, but only with around and after midgestation - prelevated tissue, with increased efficiency closer to term. Their experiment also included postnatal gonads that were amongst the most efficient in generating new follicles upon transplantation. Interestingly, the failure of pre e12.5 tissue sample to generate follicles was restricted to oocytes only, whereas granulosa cells exhibited reorganization [37].

Niikura et al. described STRA8 positive cells in the surface epithelium of ovaries of aged (20 mo old) mice, that they labeled as “quiescent germ cells”. In order to see if they are still functional, Niikura et al. transplanted senescent ovaries into young hosts and obtained GFP + immature follicles, proving there is a “reserve” of GSC that can be reactivated under appropriate circumstances. To further prove that the senescent ovary cannot sustain the folliculogenesis anymore, in spite of the existent stem cells pool, they reversed the experiment, by grafting young ovaries into senescent female mice and found a significant loss of immature follicles by 50% in 3 weeks [62].

In spite of accumulating data in favor of OSG, the controversy still exists – in 2012 Kerr et al. found no replenish of primordial follicular reserve after chemical ablation of follicles or sterilizing doses of γ-irradiation [63] and in 2013 Lei and Spradling [64] argued that folliculogenesis is sustained by very stable primordial follicles and adult ovary lack germline stem cells.

### 3. Future trends

The abnormalities of ovarian function might lead to infertility or manifestation of aggressive cancer [65]. Germ cell degeneration is observed in women affected by premature ovarian failure (POF). In addition, chemo- and radiotherapy given in cancer therapies can affect germ cell survival and cause POF and infertility [66].

Furthermore, other disorders, such as autoimmune diseases (diabetes mellitus, thyroid dysfunction, Addison disease, myasthenia gravis, Crohn's disease, lupus, or rheumatoid arthritis) and myelodysplastic syndromes, require medical treatment that can also impair reproductive cells and tissues.

Moreover, social and individual factors (lifestyle, professional career, absence of the partner, marriage in old age) have imposed delaying childbearing in women of reproductive age, affecting these women by age-related infertility. Even more so, the continuous delay of childbearing observed in developed countries will result in an increased proportion of women diagnosed with cancer before their first pregnancy [67]. Maternal ageing has detrimental effects on decidual and placental development, which may be related to repeated exposure to sex steroids and underlie the association of ageing with adverse perinatal outcomes [68].

Fertility preservation is an emerging field in medicine that enables women to maintain reproductive health and therefore, stem cells in adult human ovaries are of great interest to reproductive medicine.

Thus, oocytes and their precursor cells might be altered metabolically to sustain or increase ovarian function and fertility in women [69].

So far, orthotropic autotransplantation of cryopreserved ovarian tissue has been reported as a successful method to ensure post-chemotherapy fertility in women suffering from different malignancies [70]. The ability to isolate and promote the growth and development of such ovarian germline stem cells would provide new means to treat infertility in women. Previous studies suggest that the oogonial stem cells (OSCs) transplanted in humans could lead to functional oocytes, which would be applicable in vitro fertilization, providing virtually unlimited number of oocytes, unlike current techniques which give access to only six to eight oocytes at a time. The aim is to create a frozen source of potential oocytes for restoring fertility [71]. However, it should be noted that ovarian germline stem cells may provide oocytes in vitro, but can not form follicles in POF or aging ovaries lacking uncommitted granulosa cells [72]. Also, very recent data indicate that follicular aspirates contain a subpopulation of cells that express some stemness genes also expressed by bone-marrow derived mesenchymal stem cells, indicating their common mesodermal origin, but also have a distinct mark from both MSCs and fibroblasts. Showing also common features with granulosa cells, these cells could prove to bring useful additions to our knowledge regarding the restorative potential of ovarian follicles [7].

Translation into clinical practice and use of newly identified ovarian stem cells in infertility pathologies is a desiderate that falls in trends with the regenerative medicine concept. However, experience from other fields, such as neurodegenerative diseases, teaches us tha t is not an easily attainable goal. Closer to home, studies on spermatogonial cell line demonstrated that in vitro generation and isolation of spermatogonia is indeed possible [73-75], but with faults in their meiotic program [73], so there must be essential steps that still elude us. The oocyte-like cells express various markers consistent with oocytes such as Oct 4, Vasa, Bmp15, and Scp3. However, they remain unable to undergo maturation or fertilization due to a failure to complete meiosis [76]. To date, human OSCs have only been grown to early follicle-like structures, unacceptable for clinical use, but there are already reports of culture systems designed for in vitro human follicle development for in vitro maturation (IVM) [77]. Efforts were made to generate OSE in vitro through other means, including use of induced pluripotent cells [78], or human embryonic stem cells [79]. Same cell types were also used to generate granulosa-like cells [80].

## Conclusions

The trend of the last years seems to indicate that there is oogenesis after birth; however, the origin of the new oocytes is still unclear. Discovery of VSELs opens exciting new perspectives on tissue regeneration and regenerative medicine, but the relationship between them and new oocytes is still under debate.

## Competing interests

The authors declare that they have no competing interests.

## Authors' contributions

AA.GG: manuscript writing, conception and final design. AM.E.: collection and assembly of data, manuscript writing. ME.H: design of the study, revising the study critically for intellectual content. All authors read and approved the final manuscript.
